# Liver metastasis from papillary thyroid carcinoma treated by laparoscopic hepatectomy 43 years after resection of the primary tumor: a case report

**DOI:** 10.1186/s40792-020-00906-w

**Published:** 2020-06-18

**Authors:** Mariko Tsukagoshi, Norifumi Harimoto, Kenichiro Araki, Norio Kubo, Akira Watanabe, Takamichi Igarashi, Norihiro Ishii, Toshiki Yajima, Takaaki Sano, Ken Shirabe

**Affiliations:** 1 Department of Innovative Cancer Immunotherapy, Gunma University Graduate School of Medicine, 3-39-22 Showa-machi, Maebashi, Gunma 371-8511 Japan; 2 Division of Hepatobiliary and Pancreatic Surgery, Integrative Center of General Surgery, Gunma University Hospital, 3-39-15 Showa-machi, Maebashi, Gunma 371-8511 Japan; 3 Department of Diagnostic Pathology, Gunma University Graduate School of Medicine, 3-39-22 Showa-machi, Maebashi, Gunma 371-8511 Japan

**Keywords:** Thyroid cancer, Papillary thyroid carcinoma, Liver metastasis, Liver surgery

## Abstract

**Background:**

Papillary thyroid carcinoma (PTC) generally has a good prognosis, while liver metastasis from PTC is rare and difficult to diagnose.

**Case presentation:**

A 67-year-old woman was diagnosed with PTC and underwent a left hemithyroidectomy 43 years ago. Two years ago, thoracoscopic right middle lobectomy was performed for a lung tumor, and pathology reports confirmed a metastatic lung tumor of PTC. The patient was followed up regularly with computed tomography, and a liver tumor measuring 16 mm was found in the lateral segment of the liver. Fluorodeoxyglucose positron emission tomography (FDG-PET) was negative for liver tumor. However, FDG uptake was observed at the nodule outside the right lobe of the thyroid gland, suggesting metastasis to the right supraclavicular lymph node. Laparoscopic partial S2 hepatectomy was performed without complications. The final diagnosis was metastatic liver tumor from PTC, and the surgical margins were free of tumor. Postoperatively, the patient underwent complementary thyroidectomy and right supraclavicular lymph node dissection, followed by I-131 ablation. The final diagnosis was PTC of 7 mm and 4 mm and lymph node metastasis of 9 mm. The patient is currently doing well and has had no recurrence 1 year after surgery.

**Conclusions:**

This case demonstrates that liver metastases from PTC may be found after long-term follow-up, and liver resection might be the most appropriate treatment.

## Background

Differentiated thyroid carcinoma (DTC), including follicular and papillary carcinomas, is slow-growing tumors with good prognosis and long-term survival [1–3]. Papillary thyroid carcinoma (PTC) is the most frequent type of DTC. While PTC commonly metastasizes to regional lymph nodes, the incidence of distant metastases is rare, and the major sites of distant metastases are the lung and bone [4, 5]. Other sites of distant metastases, such as the liver and brain, are very rare, with a reported frequency of 0.5% or less [5–8]. These metastases are mostly identified during the course of treatment and follow-up, and recurrences can occur more than 30 years after initial treatment [1]. The presence of distant metastases has almost always been recognized in advanced disease and is often associated with poor outcomes [8]. Thus, early detection of distant metastases influences prognosis of the patients. Herein, we report our experience with a rare case of liver metastasis from PTC 43 years after resection of the primary tumor and review the current literature on the topic.

## Case presentation

A 67-year-old woman was referred to our department for investigation of liver tumor. The patient underwent left hemithyroidectomy for PTC 43 years prior. Two years ago, thoracoscopic right middle lobectomy was performed for lung tumor, and the diagnosis was lung metastasis of PTC. Liver tumor was found on subsequent follow-up computed tomography (CT) examinations; therefore, we considered that there was a possibility of liver metastasis of PTC.

**Fig. 1:**
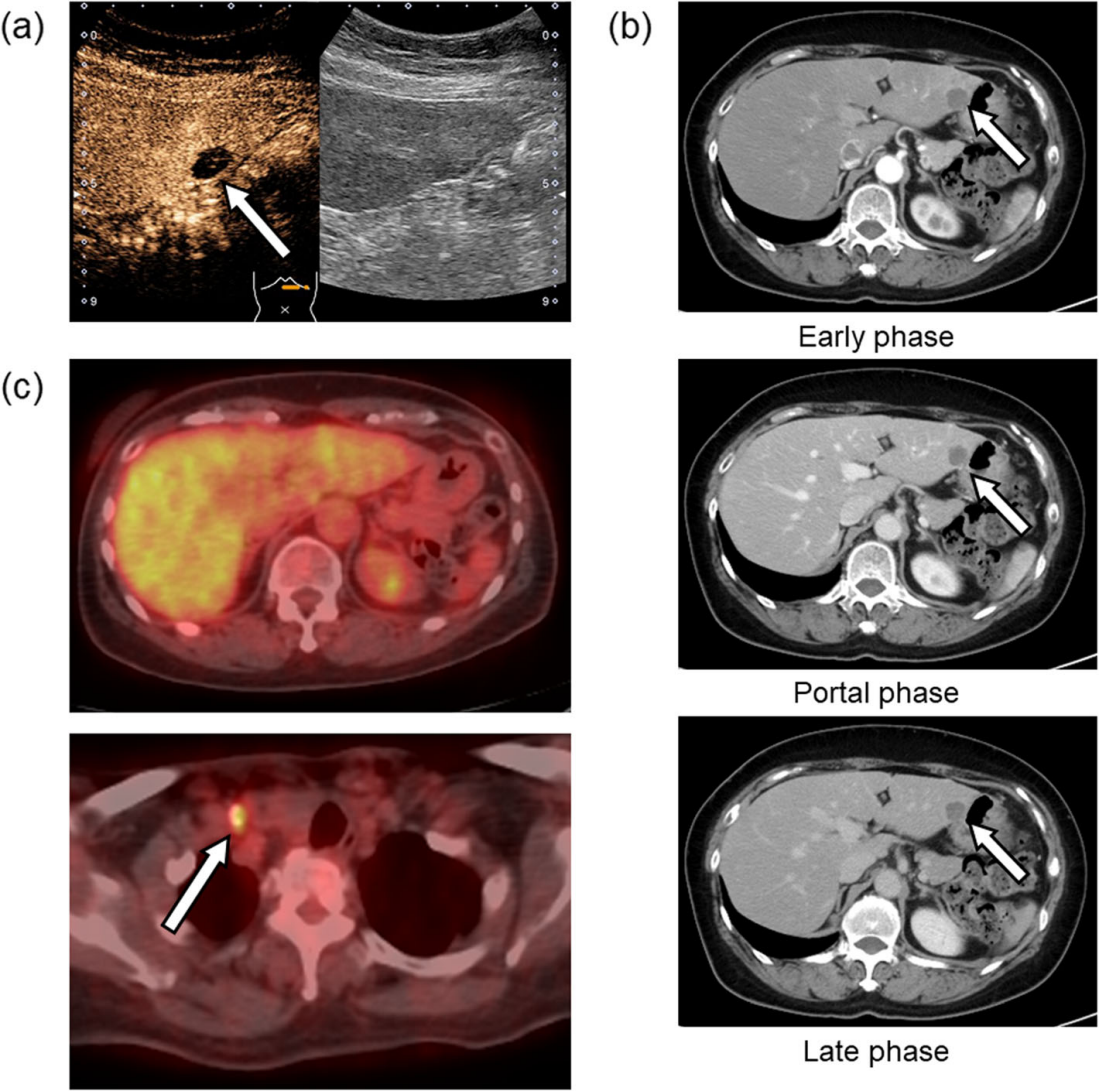
**a** Abdominal contrast-enhanced ultrasonography shows a 16-mm echo-free mass in the lateral segment of the liver, which resulted in a defect in the Kupffer phase (arrow). **b** Enhanced abdominal computed tomography scan shows a 16-mm marginally enhanced tumor in the lateral segment of the liver (arrow). **c** Fluorodeoxyglucose positron emission tomography (FDG-PET) is negative for liver tumors. FDG uptake (maximal standardized uptake value = 3.43) is observed at the nodule outside the right lobe of the thyroid gland (arrow)

Blood biochemical examinations indicated normal liver function tests, while there was no elevation of serum carbohydrate antigen 19-9, carcinoembryonic antigen, or α-fetoprotein levels. Abdominal contrast-enhanced ultrasonography showed a 16-mm echo-free image in the lateral segment of the liver and a ring perilesional enhancement from the early phase, which resulted in a defect in the Kupffer phase (Fig. 1a). An enhanced abdominal CT scan revealed a 16-mm marginal enhanced tumor in the lateral segment of the liver (Fig. 1b). The patient underwent fluorodeoxyglucose positron emission tomography (FDG-PET) which was negative for liver tumor (Fig. 1c). Approximately, half of the right thyroid gland showed no definite mass by CT. However, FDG uptake (maximal standardized uptake value = 3.43) was observed at the nodule outside the right lobe of the thyroid gland (Fig. 1c), suggesting metastasis to the right supraclavicular lymph node. The preoperative diagnosis was liver metastasis of PTC, and right supraclavicular lymph node metastasis was also suspected.

**Fig. 2:**
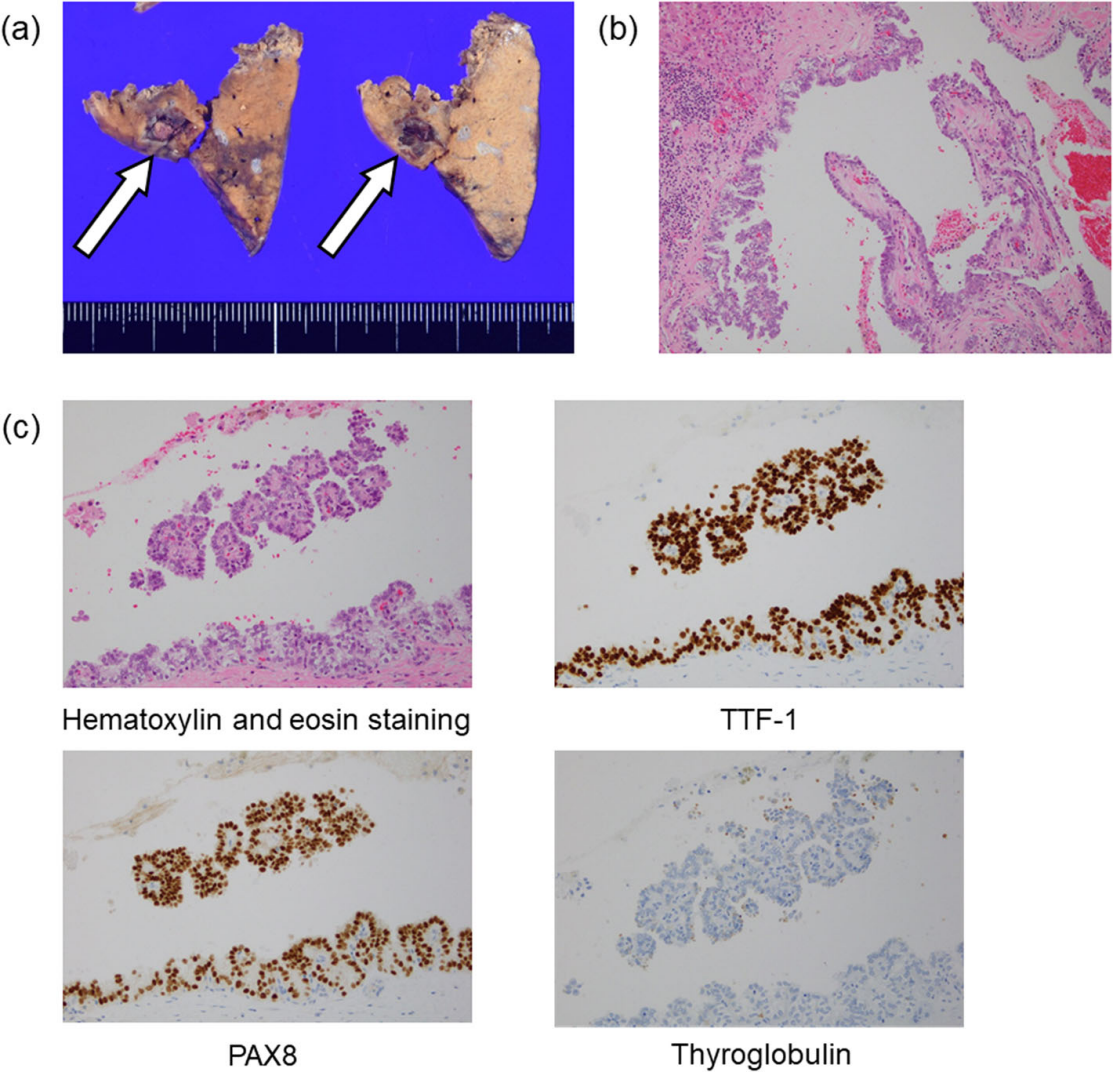
**a** Pathological finding showing a dark brown tumor (arrow). **b** Microscopically, the tumor consists of atypical cell proliferation with a papillary structure. **c** Immunohistochemically, the resected specimen revealed that the tumors are positive for thyroid transcription factor-1 and paired box 8 and slightly positive for thyroglobulin

We performed laparoscopic partial liver resection for segment 2. The operative procedures in this case and the surgical outcomes are as follows. The patient was placed in the supine position, and six ports were used including a port for Pringle taping. We placed the camera port at the umbilical site, and the operating surgeon mainly used two ports placed at the right upper abdomen. Pneumoperitoneum pressure was maintained at 10 mmHg. Ultrasonically activated scalpels and Cavitron Ultrasonic Surgical Aspirator (CUSA; AMCO Inc., Tokyo, Japan) were used for transection of the liver parenchyma. Hemostasis was performed using the tip of the CUSA connected to a VIO 300 D generator (ERBE Elektromedizin GmbH, Tubingen, Germany) and bipolar forceps held in the operator’s left hand, both at soft-coagulation mode [9]. An intermittent pedicle clamp (15 min occlusion and 5 min reperfusion, Pringle method) was used during parenchymal transection. Under anesthesia, hemodynamic management aimed to maintain low central caval pressure was used to minimize blood loss with reduced volume perfusion [10, 11]. Airway pressure was maintained between 14 and 16 cmH_2_O to reduce central venous pressure and volume perfusion [12]. The operative time was 171 min, and the volume of blood loss during the surgery was little for uncountable. The cumulative time of Pringle method was 58 min. The postoperative course was uneventful. Macroscopic examination revealed a dark brown tumor (Fig. 2a); microscopically, the tumor consisted of atypical cell proliferation with a papillary structure (Fig. 2b). Immunohistochemically, the resected specimen revealed that the tumors were positive for thyroid transcription factor-1 and paired box 8 and slightly positive for thyroglobulin (Fig. 2c). The final diagnosis was metastatic liver tumor from TPC. The surgical margins were free of tumor.

The patient was discharged 7 days after the operation without complications. Two months after surgery, the patient underwent complementary thyroidectomy (right thyroidectomy) and right supraclavicular lymph node dissection, followed by I-131 ablation. The final diagnosis was papillary thyroid carcinoma of 7 mm and 4 mm and lymph node metastasis of 9 mm. The patient has had no recurrence 1 year after surgery.

## Discussion

PTC is the most common type of thyroid carcinoma, with an occurrence of about 75 to 80% [13]. Although PTC is usually associated with favorable prognosis, the presence of distant metastases is the most significant prognostic factor and correlates with poor prognosis but is often overlooked in clinical practice [1, 5, 7, 8]. In the present case, liver metastasis was found early by CT with regular follow-up, and surgical resection could be performed.

The frequency of distant metastases in PTC is reported to be approximately 5–6% [4, 5]. Among patients with distant metastases, 45.5% occurred synchronously with the primary tumor [4]. Czepczynski et al. showed that among 1200 thyroid carcinoma patients, fewer than 1% had distant metastases detected at the time of the initial diagnosis [14]. Thus, it is relatively common for distant metastases to appear many years after the initial primary thyroid cancer surgery. Previous reports have described finding late distant metastases, other than liver metastases, more than 30 years after the initial resection of primary thyroid cancer [15, 16]. Prior to our case, only 4 cases have been reported in which liver metastases were found more than 10 years after thyroidectomy. Brient et al. reported that the mean time of onset of first liver metastases of DTC was 52 months, while the longest delay was 13 years [17]. Kouso et al. [18] reported a case of a solitary liver metastasis 32 years after curative resection of follicular thyroid carcinoma; they performed laparoscopic liver resection, but the preoperative diagnosis was primary hepatocellular carcinoma.

In the present case, the patient had lung metastasis 41 years after initial thyroid surgery and liver metastasis 43 years after initial surgery. While PTC often metastasizes to the neck lymph nodes, lungs, and bones, liver metastases are very rare and may occur with a later onset [15, 17–20]. The appearance of liver metastasis is considered to represent the terminal phase of the disease, and the prognosis is poor [7, 19, 21]. Liver metastases of DTC do not normally accumulate radioiodine because of a dedifferentiation process [19]. Therefore, scintigraphy alone during follow-up is not sufficient to detect liver metastases, and CT and/or FDG PET should also be employed [7]. In the present case, liver metastasis could be detected early by CT scan. Early detection of such distant metastases has a significant impact on treatment decisions and prognosis of the patient.

Surgery, radiotherapy, and radioactive iodine (RAI, also called I-131) ablation therapy have been used for the treatment of liver metastases from PTC. Liver resection has been reported to prolong the survival in these patients if liver metastasis is solitary [18, 22–24]. Djenic et al. advocate liver resection of solitary metastasis of DTC only if free resection margin is possible [25]. For the present case, liver metastasis of PTC could be completely resected by laparoscopic partial liver resection for segment 2. The patient had no recurrence after complementary thyroidectomy and right supraclavicular lymph node dissection followed by I-131 ablation.

Thyroid and liver diseases would interact or influence each other. The liver has an important role in thyroid hormone transport and metabolism. There is also evidence that the level of thyroid hormones may directly affect the liver structure or function [26, 27]. Regarding malignancies, some cases have been reported about the association between the thyroid and liver. Toshima et al. [28] reported a rare case of solitary thyroid metastasis from hepatocellular carcinoma detected by FDG-PET/CT.

In this case, the resected liver mass was confirmed as metastatic PTC via pathological examination and immunohistochemical analyses. Pathological examination showed two small masses in the right lobe of the thyroid. It cannot be ruled out that the primary PTC may have been present in the right lobe of the thyroid before hepatectomy; however, the patient did not present with positive signs in the thyroid by various radiological images. In cases where liver metastasis of PTC is suspected, liver resection should be considered as it offers the possibility of prolonged survival.

## Conclusions

In conclusion, liver metastases from PTC may be found after long-term follow-up of 40 years or more. Liver resection might be the most appropriate treatment of liver metastases from PTC.

## Data Availability

The datasets supporting the conclusions of this article are included within the article.

## Abbreviations

CT: Computed tomography
DTC: Differentiated thyroid carcinoma
FDG-PET: Fluorodeoxyglucose positron emission tomography
PTC: Papillary thyroid carcinoma
